# Interleukin-4 receptor alpha overexpression in human bladder cancer correlates with the pathological grade and stage of the disease

**DOI:** 10.1002/cam4.330

**Published:** 2014-09-10

**Authors:** Bharat H Joshi, Pamela Leland, Samir Lababidi, Frederick Varrichio, Raj K Puri

**Affiliations:** 1 Tumor Vaccines and Biotechnology Branch, Division of Cellular and Gene Therapies, Office of Cellular, Tissue and Gene Therapy, Center for Biologics Evaluation and ResearchNIH Building 29B, Room 2E1229 Lincoln Drive, Bethesda, 20892, Maryland; 2 Office of Biostatistics and Epidemiology, Center for Biologics Evaluation and Research, FDARockville, Maryland

**Keywords:** Biomarker, grade and clinical stage, IL-4R*α*, bladder cancer–associated protein, tumor aggressiveness

## Abstract

Previously, we have demonstrated that interleukin-4 receptor *α* (IL-4R*α*) is overexpressed on a variety of human cancers and can serve as target for IL-4 immunotoxin comprised of IL-4 and a mutated *Pseudomonas* exotoxin. However, its expression and association with grade and clinical stage of bladder cancer has not been studied. IL-4R*α* expression was examined in human bladder cancer cell lines, mouse xenografts, and biopsy specimens at mRNA and protein levels by real-time RT-PCR and IHC/ISH techniques. We also examined the effect of IL-4 on proliferation and invasion of bladder carcinoma cell lines. For tissue microarray (TMA) results, we analyzed the precision data using exact binomial proportion with exact two-sided *P*-values. We used Cochran–Armitage Statistics with exact two-sided *P*-values to examine the trend analysis of IL-4R*α* over grade or stage of the bladder cancer specimens. The influence of age and gender covariates was also analyzed using multiple logistic regression models. IL-4R*α* is overexpressed in five bladder cancer cell lines, while normal bladder and human umbilical vein cell lines (HUVEC) expressed at low levels. Two other chains of IL-4 receptor complex, IL-2R*γ*C and IL-13R*α*1, were absent or weakly expressed. IL-4 modestly inhibited the cell proliferation, but enhanced cell invasion of bladder cancer cell lines in a concentration-dependent manner. Bladder cancer xenografts in immunodeficient mice also maintained IL-4R*α* overexpression in vivo. Analysis of tumor biopsy specimens in TMAs revealed significantly higher IL-4R*α* immunostaining (≥2+) in Grade 2 (85%) and Grade 3 (97%) compared to Grade 1 tumors (0%) (*P* ≤ 0.0001). Similarly, 9% stage I tumors were positive for IL-4R*α* (≥2+) compared to 84% stage II (*P* ≤ 0.0001) and 100% stages III–IV tumors (*P* ≤ 0.0001). IL-13R*α*1 was also expressed in tumor tissues but at low levels and it did not show any correlation with the grade and stage of disease. However, the IL-2R*γ*C was not expressed. Ten normal bladder specimens demonstrated ≤1+ staining for IL-4Rα and IL-13Rα1 and no staining for IL-2R*γ*C. These results demonstrate that IL-4R*α* is overexpressed in human bladder cancer, which correlates with advanced grade and stage of the disease. Thus, IL-4R*α* may be a bladder tumor-associated protein and a prognostic biomarker.

## Introduction

Urothelial carcinoma of the urinary bladder is highly heterogeneous and the fourth most common cancer in the United States representing 72,570 (7%) of new cases diagnosed in the year 2014 [1]. Over 90% of bladder cancers (BC) are transitional cell carcinomas of urothelial origin and over 70% are non-muscle-invasive or stage Ta/T1 tumors, with the rest being muscle-invasive or stages T2–T4 [2]. Clinical manifestations including stage and grade are strongly associated with patient prognosis and play an important role in determining treatment [3]. As BC show heterogeneous clinical behavior highlighted by frequent recurrences in patients with noninvasive tumors and the potential for development of metastatic disease in those with invasive lesions, better tools to predict prognosis and refine treatment are needed. Despite intravesical BCG or chemotherapy, a majority of patients will recur and a large percentage will succumb to this cancer. Therefore, new therapeutic options are urgently needed.

IL-4, a Th2-lymphocyte-derived immunoregulatory cytokine, is one of the major cytokines responsible for differentiation of naïve CD4 T cells into the Th2 phenotype although the effector functions are mediated through a combination of IL-4, IL-13, and IL-5 [4, 5]. In preclinical models of murine lymphoma and Lewis lung carcinoma, IL-4 is shown to be a tumor growth supporting cytokine [6, 7]. It supports the cellular proliferation in human colon cancer cell lines in a concentration-dependent manner [8], whereas in pancreatic cancer IL-4 is an autocrine growth factor [9]. On the other hand, we and others have reported that IL-4 can inhibit the proliferation of human renal, colon, and breast cancer cells [10, 11] and it can cause regression of certain tumor xenografts in a mouse model of cancer [12]. It has also been shown that IL-4 receptor blockade dramatically reduces lymphatic and hematogenous metastases in vivo and thus IL-4 receptor signaling is involved in rhabdomyosarcoma tumor growth and metastasis [13].

IL-4 functions through IL-4 receptors (IL-4R). We have identified and extensively characterized overexpression of IL-4R on brain, head and neck, lung, pancreatic, AIDS-associated Kaposi's sarcoma, and prostate cancers [14–17]. In contrast, normal cells including immune cells express very low levels of IL-4R [18, 19]. The IL-4R complex exists in two different types. Type I IL-4R are composed of IL-4R*α* and IL-2R*γ* subunits (*γ*C), whereas Type II receptors have IL-4R*α* and IL-13R*α*1 subunits. Two receptor chains are required for signal transduction through type I or II IL-4R [20]. Although IL-4R are expressed in a variety of tumor cells, the significance of expression of these receptors is not known. It is also not known whether human bladder cancer expresses IL-4R and which receptor chains are present in these cells.

In this study, we examined normal bladder and BC cell lines and clinical specimens for the expression of various subunits of IL-4 receptor. We performed radio-receptor binding, real-time RT-PCR, multiplexed IHC, and ISH assays to evaluate receptor expression. BC cell lines and BC biopsy specimens in tissue microarray (TMA) showed significantly higher IL-4R*α* expression compared to normal bladder cell lines and samples. Similarly, the clinical specimens with advanced tumor grade and clinical stage showed significantly higher levels of IL-4R*α* compared to low-grade/stage disease. IL-13R*α*1 was weakly expressed in both cancer and normal cell lines and IL-2R*γ*C chain was absent in these samples. Our IHC and RT-PCR results for IL-4R expression were largely confirmed by RNAseq data from 230 human bladder cancer specimens deposited at The Cancer Genome Atlas (TCGA) database.

## Materials and Methods

### Cell lines and reagents

Human bladder cancer cell lines 5637, J82, SW780, UM-UC-3, HT-1197 were purchased from American Type Culture Collection (Manassas, VA) and primary normal bladder and human umbilical vein endothelial cells (HUVEC) cell from Lonza (Catalog # CC2533 and CC-2517; Walkersville, MD). The tumor cell lines were tested and authenticated by the vendors at the time of purchase and used within 6 months. PM-RCC renal cell carcinoma cell line was established in our laboratory and maintained in complete Dulbecco's Modified Eagle Medium (DMEM) medium, which served as a positive control cell line expressing IL-4R*α* chain.

### RNA extraction and real-time RT-PCR

Total RNA was extracted from bladder cancer cell lines using RNAEasy mini kit (Qiagen, Germantown, MD) and used to synthesize cDNA using cDNA synthesis kit (Roche, Applied Biosystem Division, Foster City, CA). Real-time RT-PCR was performed to amplify IL-4R*α*, IL-13R*α*1, and IL-2R*γ*C as described previously [21].

### ^125^I-IL-4 binding and displacement assay

Human IL-4 was iodinated with IODOGEN reagent (Pierce, Rockford, IL) according to manufacturer's instructions. For IL-4 binding and displacement assay, BC (1 × 10^6^/100 *μ*L) were incubated at 4°C with ^125^I-IL-4 (100–200 pmol/L) for 2 h with or without 200-fold molar excess of unlabeled IL-4. Cell-bound radio-ligand was separated from unbound by centrifugation through a phthalate oil gradient and radioactivity determined with a gamma counter as described previously [22].

### Cell proliferation assays

BC cells (2000 cells) were seeded in 96-well culture plates and incubated for 72 h in presence of different concentrations of IL-4. Cell growth rate was determined by MTS assay. Culture media were replaced with fresh DMEM medium containing MTS stock solution and incubated at 37°C for 2 h and absorbance was measured at 490 nm wavelength.

### Cell invasion assay

Cell invasion was studied in BC cell lines (75,000 cells) using BD BioCoat Matrigel invasion chambers (BD Biosciences, Franklin Lakes, NJ) using different concentrations of IL-4 as chemoattractant. After incubation for 48 h at 37°C, noninvasive cells were removed from the upper surface of the membrane with a cotton swab, and cells on the lower surface of the membrane were fixed and stained with 0.25% crystal violet for counting. The experiment was performed three times and five random fields/well were counted.

### Xenograft tumor development in mice

We obtained 4- to 6-week-old female athymic nude mice from the National Cancer Institute-Frederick Cancer Center Animal Facilities, Frederick, MD. Four million UM-UC-3 tumor cells were injected subcutaneously in 12 athymic nude mice and observed for tumor development. On day 10, all mice developed 20–25 mm^2^ tumors and were sacrificed. The tumors and normal tissues adjacent to the tumors were excised either for total RNA extraction or paraffin blocks for IHC analysis of IL-4R*α*, IL-13R*α*1, and IL-2R*γ*C [21]. The staining intensity was examined in a blinded fashion twice by two investigators (B. J. and F. V.) and scored as ± to 3+ independently.

### IHC and ISH analyses

IHC and ISH assays were performed for protein and RNA levels in BC and normal bladder tissue sections using IL-4R*α*, IL-13R*α*1, and IL-2R*γ*C antibodies (R&D Systems, Minneapolis, MN; and Santa Cruz Biotechnology Inc., Santa Cruz, CA) and antisense or sense riboprobes as described [21]. Tumor sections and normal/noncancerous bladder sections were scored for immunostaining in a blinded fashion. The percentage of IL-4R*α*- and IL-13R*α*1-positive fields in tissue sections was counted by viewing tissue sections under the same magnification (×200).

### Clinical specimens

Sixteen bladder cancer and 12 noncancerous/normal bladder specimens were obtained from the National Cancer Institute–supported Co-operative Human Tissue Network (CHTN) after securing approval from the U.S. Food and Drug Administration (FDA) Research Involving Human Subjects Committee (RIHSC). The histologic grading and pathology review of these samples were confirmed by one of the investigators (F. V.).

Two TMAs were purchased from commercial sources (Cybrdi, Frederick, MD; US Biomax, Rockville, MD), which consisted of 60 BC specimens with different tumor grades and 40 from the primary tumor of patients with different clinical stages and 10 normal bladder specimens.

### Statistical analysis

The results of the real-time PCR, IFA, and IHC were analyzed for statistical significance using Student's *t*-test and analysis of variance. Each experiment was repeated at least twice as described earlier. IHC analysis of TMA precision study was performed using four readings (replicates) by two investigators where each slide was read two separate times for both stage and grade. Analysis of the precision data for the four replicates was performed using exact binomial proportion with exact two-sided *P*-values. For the trend analysis to assess any change in IL-4R*α* over grade or stage levels, Cochran–Armitage statistics [23] was computed using exact two-sided *P*-values. Exact inference is a nonparametric technique, which does not require any distributional assumptions about the population of interest [24]. Multiple (or multivariable) logistic regression [25] was also performed by adjusting for age and gender in the stage database and age in the Grade database. All analyses were performed in SAS Software 9.3 (SAS Institute Inc., Cary, NC).

In addition, 230 bladder cancer specimens from TCGA was analyzed for their RNA-seq results of IL-4Rα, IL-13Rα1, and IL-2R*γ*C expression using GenePool program (Station X, Inc., San Francisco, CA, July 2014 Release). Student's *t*-test was performed to calculate *P*-values as well as *q*-values to compute false discovery rate.

## Results

### Identification and characterization of IL-4R subunits in bladder cancer cell lines

**Figure 1:**
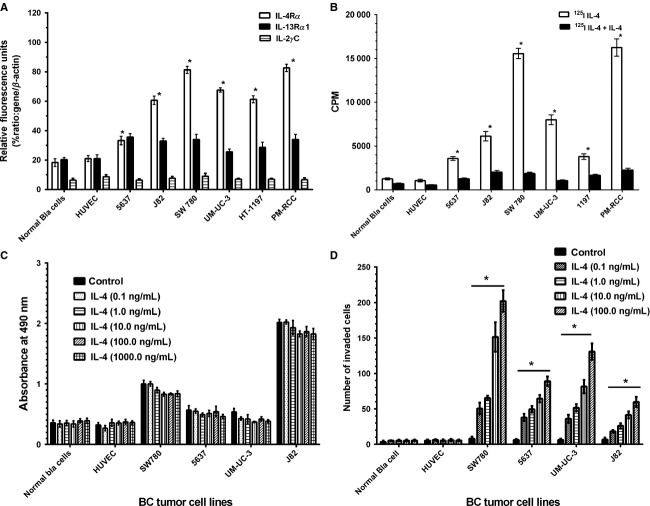
IL-4 receptor expression in bladder cancer cell lines. (A) mRNA analysis by real-time RT-PCR for IL-4R subunits in five BC, one normal bladder (Bla), one HUVEC, and PM-RCC cell lines. IL-4R*α*, white columns; IL-13R*α*1, black columns; and IL-2R*γ*C, horizontal hatched columns. Values are the mean of quadruplicate determinations; bars, SD. Results are reported in relative fluorescence units normalized to *β*-actin expression. The values are statistically significant from normal bladder and HUVEC endothelial control cell lines (*P < 0.001). The IL-4R*α* overexpressing renal cell carcinoma cell line PM-RCC was used as a positive control in these experiments. (B) Receptor binding assays for the BC cell lines. 1 × 10^6^ cells were incubated with ^125^I-IL-4 (white columns) or with ^125^I-IL-4 plus 200× unlabeled IL-4 (black columns) for competition. Bound CPM was measured in the cell pellet on a gamma counter. Data are the mean of duplicate determinations; bars, SD. The experiment was repeated three times. The values are statistically significant from normal bladder and HUVEC cell lines, which served as controls (*P < 0.001). (C) Effect of IL-4 on cell proliferation of BC cell lines. Cells, 2 × 10^3^, in 100 *μ*L complete medium were plated per well in 96-well plates and incubated in presence of different concentrations of IL-4 for 48 h. The plates were incubated for additional 2 h after addition of MTS reagent and absorbance was measured at 490 nm. (D) BC cell invasion assay: 75,000 cells in 750 *μ*L of serum free medium were plated in upper chamber and different concentrations of IL-4 in 500 *μ*L of serum-free medium were used as chemoattractant in lower chamber. Numbers of invaded cells were counted after staining with crystal violet stain. Data are the means of quadruplicate determinations; bars, SD. Each bar represents the number of invaded BC cells treated with different concentrations of IL-4. Controls cells received same volume of PBS. The values are statistically significant between controls and IL-4-treated BC cell lines (*P < 0.001).

Five human bladder cancer, one normal bladder, and one HUVEC endothelial control cell lines were studied for expression of the three subunits of IL-4R at mRNA and protein levels. Real-time RT-PCR showed significantly higher mRNA expression for IL-4R*α* in all BC cell lines compared to normal bladder and HUVEC cell lines (*P* ≤ 0.001) (Fig. 1A). The IL-13Rα1 was also expressed in all cell lines including normal bladder but expression was at lower levels compared to IL-4R*α*. The expression of IL-2R*γ*C was below the range of detection in all samples.

We performed binding studies to determine levels of IL-4R expression using radio-labeled IL-4. All cell lines specifically bound ^125^I-IL-4, as binding was competed by 200-fold excess of unlabeled IL-4 (Fig 1B). However, BC cell lines showed highest binding compared to normal bladder and HUVEC endothelial control cell lines (*P* < 0.001).

### Effect of IL-4 on cell proliferation and invasion

We examined the effect of IL-4 on proliferation of four BC cell lines. IL-4 showed only modest growth inhibition which was not statistically significant (Fig 1C). When the invasion ability of each BC cell line was examined using a matrigel invasion assay, IL-4 significantly increased the cell invasion in all four BC cell lines compared to respective control (*P* < 0.001) (Fig. 1D). The highest number of invaded cells was observed in SW780 followed by UM-UC-3, 5637, and J82 cell lines. On the other hand, IL-4 had no effect on normal bladder and HUVEC cell lines.

### Characterization of IL-4R in BC xenografts

**Figure 2:**
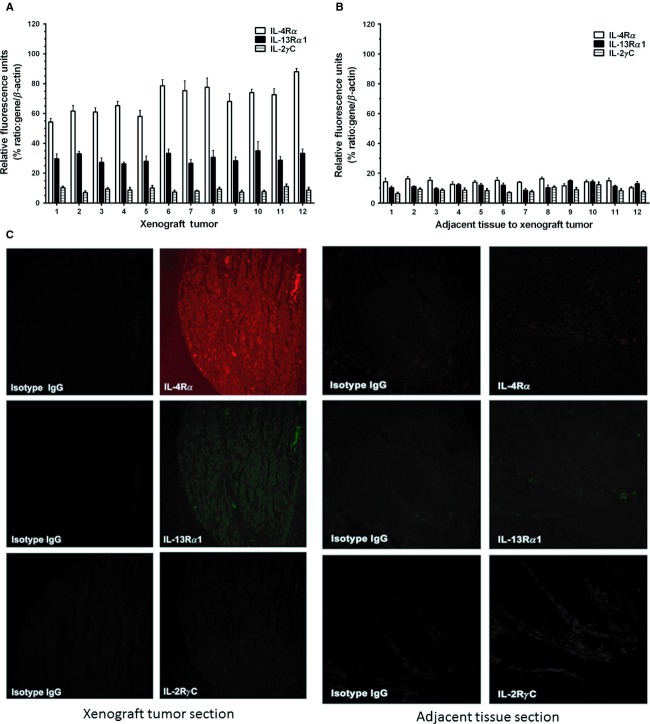
IL-4R expression in xenograft tumors. mRNA analysis by real-time RT-PCR for IL-4R subunits in 12 xenograft tumors developed from UM-UC-3 BC cells in athymic nude mice (A) and in normal tissues adjacent to xenograft tumors (B). IL-4R*α*, white columns; IL-13R*α*1, black columns; and columns with horizontal hatches, IL-2R*γ*C. Columns, mean of determination; bars, SD. IHC staining of xenograft tumor and adjacent tissue section for IL-4R*α*, IL-13R*α*1, and IL-2R*γ*C chains as described in Materials and Methods (C).

To determine whether human IL-4R expression is maintained in xenografts, we examined mRNA expression by real-time RT-PCR for IL-4R*α*, IL-13R*α*1, and IL-2R*γ*C. These bladder tumor specimens showed three to six times higher expression of IL-4R*α* mRNA compared to adjacent tissue specimens in all 12 samples (*P* ≤ 0.001). IL-13R*α*1 mRNA was also expressed but at much lower levels (*P* ≤ 0.01) and IL-2R*γ*C mRNA was marginally expressed in these specimens (Fig 2A and B). IHC analyses from tumor and adjacent specimens corroborated the mRNA results and demonstrated overexpression of IL-4R*α* and IL-13R*α*1 in tumor compared to surrounding tissue. None of these specimens showed positive immunostaining for IL-2R*γ*C chain (Fig. 2C).

### In situ expression of IL-4R chains in BC biopsy specimens

**Figure 3:**
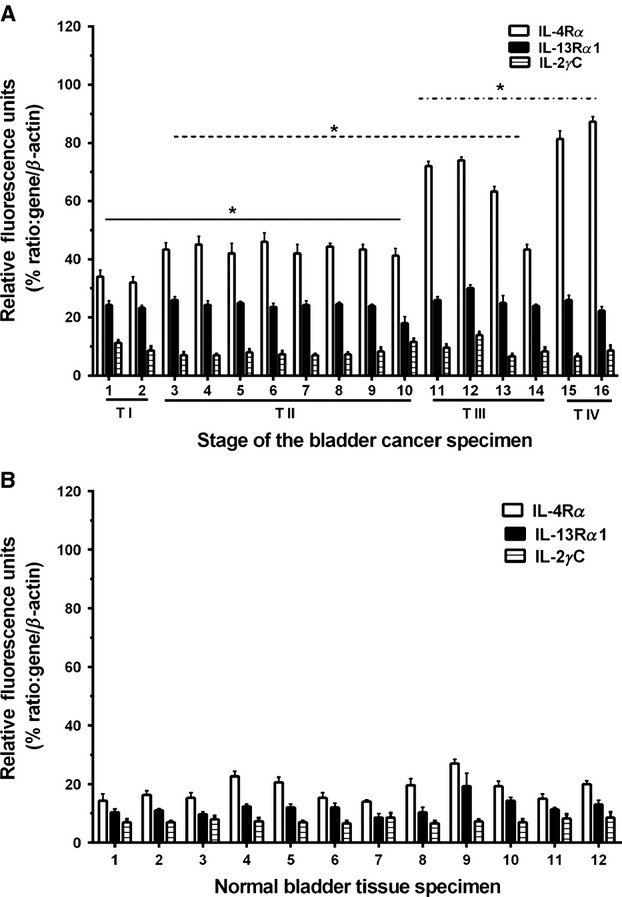
Analysis of IL-4R expression in BC biopsy samples. mRNA analysis by real-time RT-PCR for IL-4R subunit in 16 biopsy specimens of human BC (A) and 12 normal bladder specimens (B). mRNA was extracted from paraffin blocks and amplified as described in Materials and Methods. IL-4R*α*, white column; IL-13R*α*1, black column; and column with horizontal hatches, IL-2R*γ*C. Columns, mean of determination in BC specimens, or normal bladder specimens. The values are statistically significant among TI and TII, TII and TIII, and TIII and TIV (*P < 0.001).

A total of 28 specimens, which consisted of 16 tumor specimens and 12 normal/noncancerous bladder specimens obtained from CHTN were analyzed for mRNA expression of IL-4R*α,* IL-13R*α*1, and IL-2R*γ*c by real-time RT-PCR assay (Fig. 3A). Two of 16 tumor specimens were stage I (TI), eight were stage II (T II), four were stage III (T III), and two were stage IV (T IV). We observed that mRNA expression for IL-4R*α* was significantly higher in all tumor specimens especially those with advanced clinical stage disease compared to 12 normal/noncancerous bladder specimens (Fig 3A and B). Although the number of specimens in stages I and IV was small, we observed a trend of increasing IL-4R*α* mRNA expression with increasing clinical stage of disease. IL-4R*α* mRNA expression was significantly higher ranging between 33 and 88% relative fluorescence units (RFU) in tumor specimens (*P* ≤ 0.001) compared to 12.5–22% in normal/noncancerous bladders specimens. Interestingly, RFU values in BC biopsy specimens were higher in T II than T I, T III than T II, and T IV than T III (*P* ≤ 0.001, Fig 3A). In contrast, IL-13R*α*1 mRNA was expressed but at lower levels of RFUs of 22–28% in tumor specimens compared to 8–10% in normal/noncancerous bladder specimens (Fig. 3B) (*P* ≤ 0.001). RFU values for IL-2R*γ*C mRNA in real-time RT-PCR analysis were found to be similar in tumor and normal/noncancerous specimens ranging between 7% and 10%.

IHC analyses of biopsy specimens for each subunit of IL-4R showed immunostaining for IL-4R*α* that ranged between 1+ and ≥3+ (Table 1). Two of 16 tumor samples showed very strong staining (4+), six strong staining (≥3+), eight moderate staining (2+), and two samples 1+ staining. In contrast, normal/noncancerous bladder specimens showed barely detectable staining for IL-4R*α* (<1+). The IL-13R*α*1 staining was weak to modest (<1+) in tumor and normal/noncancerous specimens suggesting weak or no overexpression of IL-13R*α*1 chain in BC specimens (Table 1). Antibody to IL-2R*γ*C and isotype control (IgG) showed no staining in these specimens suggesting that this chain is either absent or below detection limits of the assay, which corroborated with the real-time RT-PCR data.

**Table 1:** IHC and ISH analysis for IL-4R subunits in bladder specimens.

	Bladder cancer specimens	Normal/noncancerous bladder specimens
No	16	12
Male	9	7
Female	7	5
Age	44–66	38–66
*IL-4Rα*		
IHC		
±	0 (0/0)	3 (6–8)
1+	2 (35–38)	9 (4–15)
2+	8 (28–75)	0 (0/0)
3+	4 (46–78)	0 (0/0)
4+	2 (36–56)	0 (0/0)
ISH		
±	0 (0/0)	2 (4–7)
1+	2 (22–26)	10 (2–9)
2+	11 (20–70)	0 (0/0)
3+	3 (46–70)	0 (0/0)
*IL-13Rα1*		
IHC		
±	4 (4/12)	3 (4–8)
1+	12 (10–34)	9 (4–15)
ISH		
±	3 (12–16)	1 (2)
1+	13 (6–24)	11 (2–9)

Extent of IHC or ISH was evaluated as ±, weakly positive; 1+, moderate; 2+, strong; 3+ stronger; 4+, strongest. Percent positive field was counted after viewing at 200× magnification and shown in the parenthesis.

The tissue specimens were also hybridized in situ (ISH) with sense and antisense ribo-probes for detection of IL-4R*α* and IL-13R*α*1 mRNA. Similar to IHC results, three BC specimens showed strong hybridization for IL-4R*α* (3+), while 11 samples showed moderate (2+) hybridization. IL-13R*α*1 hybridization was weak to modest (<1+) in all samples. In contrast, normal bladder/noncancerous specimens showed weak to moderate hybridization for both chains (Table 1), corroborating IHC and real-time RT-PCR results.

### Association of IL-4R*α* expression with advanced pathologic grade and clinical stage of bladder cancer

We further investigated the expression of IL-4R subunits in TMA specimens from two commercial vendors. The intensity of IHC staining was analyzed by two investigators in a blinded fashion for statistical significance.

For the precision study of the Grade database, 60 BC specimens were read by two investigators on two different occasions resulting in four different readings to score IL-4R*α* and IL-13R*α*1 intensity. Similarly, 40 BC specimens in the stage database were also read to generate four readings for each slide. Scores of ≥2+ were considered positive, while ≤1+ were considered negative. There was a very high concordance among the four readings in both databases, where scores 3+ and 4+ were combined as 3+, while scores 1+ and no score (value ±) were combined as 1+. There was a very high concordance among the four readings in both databases, where 55/61 (90.2%) specimens had concordant scores (exact 95% CI = 79.8–96.3) for the Grade database and 43/50 (86%) specimens had concordant scores (exact 95% CI = 73.3–94.2) for the stage database. These results showed high precision in the IHC measurements for both databases. The discordant scores were resolved by assigning the majority score. For the remainder of the analysis, discordant scores were resolved by assigning the majority score and when there was an equal number of discordant scores, the lower score was used. For the rest of the analysis, all discordant cases in IL-4R*α* were coded as negative. The expression for IL-13R*α*1 was not observed in either TMA databases as the expression for IL-13R*α*1, was found to be ≤1+ in all specimens.

**Figure 4:**
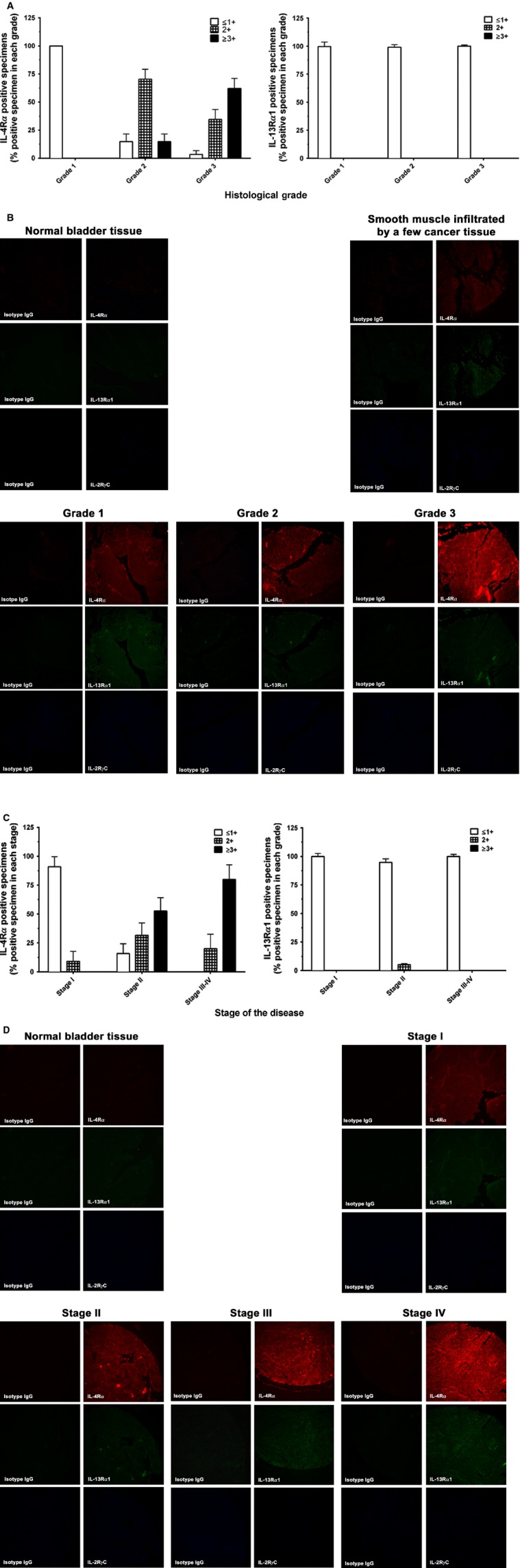
Patterns of IL-4R expression observed in various pathologic grade and clinical stage of BC Specimens by IHC. BC specimens with pathologic grades 1, 2, 3 and tumor stages I, II, III, and IV were stained with either isotype control or anti-IL-4R*α*, anti-IL-13R*α*1, and anti-IL-2R*γ*C antibodies as described in Materials and Methods. IL-4R*α* was detected using alexa 545-conjugated anti-mouse IgG for red fluorescence, IL-13R*α*1 using alexa 488-conjugated anti-goat IgG for green fluorescence, and IL-2R*γ*C using AMCA-conjugated anti-rabbit IgG for blue fluorescence. Percent positive numbers of specimens with pathological grade are shown for positive staining of IL-4R*α* and IL-13R*α*1 (A) and a representative IHC analysis of a specimen from each pathological grade is shown in (B). Similarly, percent positive number of specimens in each clinical stage is shown with immunostaining intensity for IL-4R*α* and IL-13R*α*1 (C) and representative IHC analysis of these chains in specimen from each stage (D). IHC analysis was performed and fields were counted in blinded fashion at ×200 magnification.

IHC results show that IL-4R*α* expression is associated with a substantial increase in the proportion of subjects as the grade advances from 1 to 3 for both IL-4R*α* values (2+ and 3+). A sum of the positive values of IL-4R*α* (2+ and 3+ combined) showed a statistically significant increasing trend with the pathological grade (grade 1 = 0%, grade 2 = 85.2%, grade 3 = 96.6%; Exact *P* < 0.0001) compared to negative values of IL-4R*α* (1+). The significant increasing trend was also observed even after adjusting for age (*P* = 0.0025) in this cohort (data not shown). IL-13R*α*1 expression in all grades was found to be similar to its trend observed in specimens (Fig 4A). In contrast, 10 normal bladder specimens showed ≤1+ staining for both IL-4R*α* and IL-13R*α*1 chains. In addition, none of the specimens showed any appreciable staining for IL-2R*γ*C chain in either tumor or normal bladder TMAs (Fig 4B).

Similarly, IHC analysis of BC specimens with different stages demonstrated a substantial increase in the proportion of patients as stage advanced from stages I to III–IV for both IL-4R*α* values of 2+ and 3+. Interestingly, the positive values of IL-4R*α* (≥2+ and 3+ combined) in comparison to negative values of IL-4R*α* (≤1+) showed a statistically significant increasing trend with the stage of disease (stage I = 9.1%, stage II = 84.2%, stages III–IV = 100%; Exact *P* ≤ 0.0001). This trend was maintained even after adjusting for age and gender (*P* = 0.002). We did not observe an increasing trend of IL-13R*α*1 expression in these specimens. All specimens showed weak staining (≤1+) for IL-13R*α*1 chain (Fig 4C). As shown in Figure 4D, IHC staining of a representative normal, stage I, II, III, and IV tumor for IL-4R*α*, IL-13R*α*1, and IL-2R*γ*C chain demonstrated an increasing trend of IL-4R*α* immunostaining. IL-13R*α*1 staining was moderately positive compared to normal bladder specimen but did not show a dramatic increase similar to IL-4R*α* chain in BC tumor specimens.

In addition, we analyzed mRNA gene expression of three subunits of the IL-4R complex using RNA-seq data deposited at the TCGA database using GenePool software. TCGA database has RNA-seq data from 230 human bladder cancer specimens. TCGA data analyses showed that IL-4R*α* gene was expressed in bladder cancer samples. The higher stage specimens showed higher level of gene expression compared to low-stage tumors. These data corroborated with our RT-PCR and IHC analyses results. However, the difference of gene expression between high-stage and low-stage tumors was not statistically significant due to only two specimens of stage I tumors were deposited at this data base. The gene expression for IL-13R*α*1 and IL-2R*γ*C also corroborated with our IHC and RT-PCR results. The mRNA for IL-13R*α*1 was expressed at low levels and its expression was similar in all stages of cancer. The expression of IL-2R*γ*C mRNA was undetectable to a very low level detection. When gene expression data for IL-4R*α* were compared between the grade of bladder cancer, the TCGA data analyses showed that lower stage disease has higher gene expression compared to high-grade specimens (*P* < 0.03) and fold difference of 1.35. While these data for IL-4R*α* do not support our real-time RT-PCR and IHC results but expression of IL-13R*α*1 and IL-2R*γ*C corroborated with our results.

## Discussion

Several previous studies have demonstrated overexpression of IL-4R*α* in a variety of human cancers, for example, pancreatic adenocarcinoma, renal cell carcinoma, ovarian carcinoma, AIDS-associated Kaposi sarcoma, glioblastoma, squamous cell carcinoma of head and neck, colon carcinoma, gastric carcinoma, breast carcinoma, and lung carcinoma [19]. However, the significance and relationship between IL-4R*α* expression and disease aggressiveness have not been established. We have developed an IL-4R-targeted immunotoxin to take advantage of overexpression of IL-4R in different types of human cancers. This recombinant immunotoxin consists of a circularly permutated IL-4 and a mutated form of *Pseudomonas* exotoxin [termed IL4 (38-37)-PE38KDEL, cpIL4-PE], which shows highly specific cytotoxicity to IL-4R*α*-positive tumor cells in vitro [17, 26, 27]. In xenograft models of human cancer, cpIL-4PE has been shown to mediate remarkable antitumor activity [14, 26, 28]. Based on these and other preclinical studies, several phase 1 clinical trials were completed. These studies indicate that cpIL-4PE immunotoxin can be safely administered intracranially while causing necrosis of glioma with little toxicity to normal adjoining brain tissues [29, 30]. In addition, cpIL-4PE immunotoxin can be safely given intravenously at various doses to a maximum tolerated dose [31].

As it was not known whether human BC expressed IL-4R, we examined the expression of these receptors in cell lines and pathological samples for their potential role as *bladder tumor-associated protein*. We also examined a possible relationship between IL-4R expression and grade and stage of disease. We demonstrate that IL-4R*α* is overexpressed in BC cell lines. We also demonstrate that IL-13R*α*1 is expressed but at low levels, while IL-2R*γ*C is undetectable or below the detection limits of the assay. In contrast, normal bladder and HUVEC cell lines showed only marginal expression for IL-4R*α* and IL-13R*α*1 chains and undetectable IL-2R*γ*C. Overexpression of IL-4R*α* was also demonstrated by IHC in tumors but not in normal bladder samples confirming the data in the BC cell lines. Based on these results, we identify that human bladder cancer express type II IL-4R.

To confirm our results, we accessed NCI's TCGA database, TCGA Research Network (cancergenome.nih.gov), NCBI Gene Expression Omnibus data repository (http://www.ncbi.nlm.nih.gov.geo/) and the COSMIC/Sanger data base (*cancer.sanger.ac.uk)* [32, 33]. While these databases provide data from various tumors, none of these databases have published gene expression data from bladder cancer or IL-4R*α* expression. Whole-genome sequencing of tumors and matched normal samples focused on 18,091 genes in urothelial cancers, but it did not include IL-4R*α* [32, 34]. Additional data analysis of RNA-seq results from a TCGA subset of 230 bladder cancer specimens by GenePool program largely confirmed our RT-PCR and IHC results for all three chains of IL-4R. However, RNAseq revealed a 1.35-fold difference for IL-4R*α* expression in low-grade bladder cancer specimens compared to high-grade specimens (*P* ≤ 0.03). But, it showed a nonsignificant trend of IL-4R*α* overexpression between stages III and IV versus stages II and I tumors. The discrepancy between our results for IL-4R*α* expression by real-time RT-PCR and IHC and RNAseq of TCGA may be due to many technical and analytical reasons. It is known that the cDNA library construction for RNA-Seq is complex and may introduce bias, which can misrepresent the gene expression levels at every step of the library construction (such as reverse transcription, adaptor addition, amplification, etc.). In addition, it is also known that the accuracy for RNA-Seq depends on the sequencing depth, replicate numbers, and the sequencing platform, all of these may introduce some bias. Finally, there is no gold standard data analysis software for RNA-seq analysis; available softwares have their own strength and limitations to identify differentially expressed genes. On the other hand, real-time RT-PCR and IHC results are not confounded by mass data analysis as RNAseq data. It is hoped that the future datasets will be available that include IL-4R expression to validate our results.

Expression of IL-4R*α* in bladder cancer appeared to correlate with advanced grade of disease. IHC analyses for IL-4R subunits in TMA with 60 samples revealed that a significant number of tumor samples with advanced epithelial BC grade 2 and 3 disease showed an intense immunostaining for IL-4R*α* compared to grade 1 disease, while IL-13R*α*1 expression was marginal and not different between grades of the disease. The reason for this difference in IL-4R*α* expression between grade 2/3 and grade 1 BC samples is not known. However, it appears that IL-4R*α* may be upregulated in advanced grade of tumors. A recent study reported tumor expression of IL-4R*α* is inversely correlated with survival in patients undergoing surgical resection for epithelial malignant pleural mesothelioma [3].

The second set of TMA of 40 specimens with different clinical stages showed significant overexpression of IL-4R*α* in tumor samples with advanced stage disease. IL-4R*α* expression was associated with tumor cells but not infiltrating cells including macrophages or stromal cells. To our knowledge, this is the first study to demonstrate that IL-4R*α* chain may act as a bladder tumor-associated protein and a relationship between its expression and tumor grade and stage of BC disease.

The significance of IL-4R expression in human cancer and in bladder cancer is not clear. IL-4 is known for its differential positive and negative effects on tumors, which may depend on whether IL-4 is delivered exogenously or produced endogenously [35]. We observed that exogenous IL-4 could only modestly inhibit the growth of bladder cancer cell lines in vitro. A study in mice showed that IL-4R*α* competent murine tumors exhibited increased tumor growth when compared with tumors from IL-4R*α*-deficient counterparts [36]. Tepper et al., have shown that T cells, B cells, NK cells, and mast cells are not required for the IL-4 induced protective immunity, because no tumor have grown in nu/nu (deficient of T cells), SCID (deficient of T and B cells), bg/bg (deficient of NK cells), w/wv (deficient of mast cells) mice that received *s.c*. inoculation of IL-4-producing plasmacytoma, J558L or melanoma B16 cells [37]. In contrast, Hock et al., have reported the growth of plasmacytoma tumor in T-cell-deficient mice, including nu/nu, SCID/beige (deficient of T, B, and NK cells), and NIH III mice (also deficient of T, B, and NK cells) after a long latency of 30 days [38]. In immunocompetent mice, no plasmacytoma tumor growth was observed. Cell depletion study indicated that CD8+ T lymphocyte were important, whereas CD4+ T cells played only a marginal role in this model [38].

Interestingly, Todaro et al., have shown that resistance to drug-induced apoptosis in CD133+ colon cancer cells was mediated through increased production of IL-4 [39]. In murine breast cancer models, IL-4 secretion by CD4+ Th2 cells activate tumor-associated macrophages (TAM) to facilitate pulmonary metastasis mediated by secretion of EGF [40]. In addition, IL-4 is found to promote tumor metastasis in B16 melanoma mouse model. High metastatic variant of B16 melanoma (B16F10) induced a CD4+ T-cell population with strong IL-4 production compared to T cell induced by the low metastatic variant B16F1. Systemic IL-4 administration increased the metastasis of B16F1 to the level of B16F10 and neutralizing antibody to IL-4 decreased the metastasis in these cell lines to promote cell proliferation [41]. Consistent with above reports, we also found that IL-4 can mediate significant invasion of BC cells in vitro.

IL-4 and IL-4R may also be involved in modulating Th1 and Th2 T-cell responses to cancer. In fact, many clinical studies report abnormal Th1 to Th2 ratio in patients with various cancers and it has been shown that the nature of immune response can affect outcome in patients with renal cell cancer, non–small lung cancer, prostate cancer, colon cancer, and other types of cancers [42, 43]. Specifically, IL-4-producing T cells are elevated in patients with cancers that parallel worse prognosis [44–46]. It has been shown that the natural immune reaction to human malignant pleural mesothelioma involved tumor infiltrating T cells which are skewed toward an IL-4-producing phenotypes [47]. Interestingly, Zelba et al., demonstrated a negative effect on survival in stage IV melanoma patients with circulating CD4+ T cells producing IL-4 and IL-17 upon Melan A stimulation [48]. Similar observations were also reported previously that patients with Melan A-reactive CD4+ T cells producing IL-4 had mean survival time of only 6 months compared to 12 months for patients without IL-4 responses in a phase II clinical trial of recombinant human IL-4 in patients with disseminated malignant melanoma [49]. Recently, Volonte et al., showed that cancer-initiating cells (CIC)-associated membrane IL-4 and IL-4R mediated the inhibition of T-cell proliferation in coculture of PBMCs from colorectal cancer patients or healthy donors with autologous or allogeneic CICs. Cell-to-cell contact was required as cocultivation without the cellular contact of T lymphocytes with CICs or with CIC-derived cell culture supernatants did not affect their proliferation. Thus, blocking of IL-4 may affect T-cell-mediated tumor activity, which could lead to a skewed immune response toward T-cell effector responses [50].

Our present data suggest that the IL-4/IL-4R*α* axis may be a promising target for various therapeutic approaches for BC therapy. Potential agents may include blockers of IL-4 binding to its receptors and agents that bind IL-4R*α* and mediate cytotoxicity to cells, such as cpIL-4PE immunotoxin. Additional studies are ongoing to explore these possibilities. Our findings also demonstrate that IL-R*α* overexpression in BC tumors can function as a newly identified bladder tumor-associated protein or biomarker involved in cancer pathogenesis, and may characterize disease aggressiveness. Finally, these results suggest that IL-4R*α-*targeted therapies would be more appropriate for advanced grade/stage tumors.
